# Composite GDP nowcasting using macroeconomic variables and electricity data

**DOI:** 10.1371/journal.pone.0324381

**Published:** 2025-06-09

**Authors:** Zhiqiang Lan, Zidi Liu, Guoyao Wu

**Affiliations:** 1 State Grid Fujian Marketing Service Center (Metering Center), Fuzhou, China; 2 Department of Statistics and Data Sciences, Xiamen University, Xiamen, China; 3 Laboratory of Digital Finance, Xiamen University, Xiamen, China; ITAM, MEXICO

## Abstract

Accurate and timely forecasting of the gross domestic product (GDP), known as “nowcasting”, is crucial for macroeconomic regulation. In this paper, we propose a composite GDP nowcasting model that combines predictions from a dynamic factor model using mixed-frequency macroeconomic indicators and a regression model using real-time electricity data. In the regression model, we introduce changes in electricity capacity, in addition to electricity consumption, as a new predictor to reflect expectations for future electricity demand. Such a nowcasting model not only leverages the correlation between GDP and other macroeconomic indicators, but also utilizes the information contained in electricity data, which is closely related to production. We perform nowcasting for year-on-year quarterly GDP growth rates using data from Fujian Province, China. The results demonstrate that the proposed composite nowcasting model effectively reduces forecast errors compared to the dynamic factor model and the regression model.

## 1 Introduction

Gross domestic product (GDP) is a core indicator in national economic accounting. It provides essential references for decision-making by the government and enterprises. However, due to the complexity of GDP accounting processes, it is typically released quarterly with substantial delays. To address this issue, many nowcasting methods have been developed to utilize high-frequency and real-time information to predict current or near-term GDP. Parigi and Schlitzer [1] proposed the bridge model for GDP nowcasting to mitigate the issue of data release delays. They constructed bridge equations to forecast quarterly-released national account variables, including GDP, using aggregated monthly economic indicators. When the monthly indicators for the entire current quarter have not been released, they suggested using time series models, such as the ARIMA model, to forecast the unpublished monthly data. However, the bridge model may loss information when aggregating the monthly data.

To avoid the shortcomings of the bridge model, scholars have proposed a variety of nowcasting models that utilize mixed-frequency data. The Mixed Data Sampling (MIDAS) model incorporates mixed-frequency variables into a unified regression model by setting a weighting function to avoid information loss caused by temporal aggregation [2, 3]. Furthermore, Gu$\overset{´}{\text{e}}$rin and Marcellino [4] developed the Markov Switching Mixed Data Sampling (MS-MIDAS) model for forecasting U.S. GDP. Different from the MIDAS approach, the Mixed-Frequency Vector Autoregression (MF-VAR) model transforms low-frequency data into high-frequency data using state-space models and subsequently predicts GDP growth rates [5, 6]. Kuzin *et al*. [7] and Fang *et al*. [8] have found that the MIDAS performs well in short-term forecasts, while the MF-VAR is more effective for long-term forecasts. The MIDAS and MF-VAR may suffer from the “curse of dimensionality" when many predictor variables are involved. Stock and Watson [9] argued that macroeconomic fluctuations are dominated by unobservable common factors. Building on this insight, the dynamic factor model (DFM) has been developed to address the issue of introducing an excessive number of model parameters [10, 11]. The forecasting accuracy and sensitivity of the DFM have been verified in numerous applications. Liebermann [12] employed the DFM for U.S. GDP forecasting and found that the forecast accuracy improved with the use of real-time released data, outperforming the results from professional forecaster surveys. Chikamatsu *et al*. [13] applied the DFM to forecast Japanese GDP, demonstrating its superior performance in forecasting accuracy. Hindrayanto *et al*. [14], Reijer and Johansson [15] confirmed that the DFM can capture significant economic fluctuations, such as financial crises.

It is worth noting that, in addition to various macroeconomic indicators containing information about GDP trends, electricity consumption data, which is closely related to production, can also reflect short-term changes in the economy. Numerous studies have shown that there is a strong correlation between energy consumption and economic growth [16–18]. Shiu and Lam [19] indicated that GDP and electricity consumption are cointegrated in China and there is an unidirectional Granger causality from electricity consumption to real GDP. Ciarreta and Zarraga [20] showed evidence of a strong causality from electricity consumption to GDP in a set of 12 European countries. These findings indicate that real-time electricity data can play a significant role in GDP nowcasting.

In this paper, we use macroeconomic indicators and electricity data from Fujian Province, China, to forecast its quarterly GDP growth rate. We implement a three-step process to integrate information from both macroeconomic indicators and electricity data. Firstly, we employ a dynamic factor model for mixed-frequency macroeconomic indicators to forecast the GDP growth rate. Secondly, we utilize real-time electricity data for GDP nowcasting. Specifically, we incorporate not only electricity consumption data but also include changes in electricity capacity as a predictor variable. The change in electricity capacity reflects the production side’s expectations for future electricity demand, which can enhance the accuracy of GDP forecasting. Finally, we use model averaging to combine GDP predictions based on economic indicators with those based on electricity data.

The remainder of this article is organized as follows. Sect 2 introduces the nowcasting process, including construction and estimation of both the dynamic factor model and the regression model that incorporates electricity data, as well as the method of model averaging. Sect 3 uses macroeconomic indicators and electricity data from Fujian Province to forecast its quarterly GDP growth rate, demonstrating the effectiveness of our proposed nowcasting procedure. Sect 4 concludes the paper.

## 2 Composite GDP nowcasting method

This section introduces our nowcasting model for the year-on-year growth rate of quarterly GDP. Let *t* be the index for month and *l* be the index for quarter. Then t=3(l−1)+k, where k=1,2,3 and l=1,2,⋯, denotes the *k*-th month in quarter *l*. Furthermore, we denote the vector of *n* monthly variables by $y_{t}^{M}=(y_{1,t}^{M},\cdots ,y_{n,t}^{M}{)}^{T}$, which includes various monthly economic indicators. We also denote the vector of *m* quarterly indicators by $y_{t}^{Q}=(y_{1,t}^{Q},\cdots ,y_{m,t}^{Q}{)}^{T}$, where $y_{1,t}^{Q}$ specifically represents the year-on-year growth rate of quarterly GDP. For simplicity, we assume that all indicators are standardized so that their means are zero and their variances are one. The vector of monthly variables, $y_{t}^{M}$, is observed every month, while the vector of quarterly variables, $y_{t}^{Q}$, is only observed for *t* = 3*l*. An exception is $y_{1,t}^{Q}$, the quarterly GDP data, which is released with a delay of approximately 20 days. We consider predicting $y_{1,3l}^{Q}$, the year-on-year growth rate of quarterly GDP, at times t=3l−2, t=3l−1, and t=3l, respectively.

### 2.1 Dynamic factor model

In this paper, we consider the dynamic factor model as the baseline model. More specifically, we introduce 15 macroeconomic indicators in addition to GDP into our model, as shown in Table 1, among which 13 are monthly variables and 2 are quarterly variables. These 15 indicators are divided into 6 categories: trade indicators, industrial indicators, consumption indicators, logistics indicators, policy indicators, and power indicators, which is also presented in Table 1.

**Table 1:** List of macroeconomic indicators and their common factor categories.

Predictive indicators	Frequency	Common factor categories
Gross Domestic Product (GDP)	Quarterly	-
Import amount	Monthly	Trade indicator
Export amount	Monthly	Trade indicator
Value added of industries above designated size	Monthly	Industrial indicator
Operating revenue industrial enterprise	Monthly	Industrial indicator
Producer price index (PPI)	Monthly	Industrial indicator
Per capita disposable income of urban residents	Quarterly	Consumption indicator
Consumer price index (CPI)	Monthly	Consumption indicator
Total retail sales of consumer goods	Monthly	Consumption indicator
Fixed asset investment	Monthly	Consumption indicator
Highway freight volume	Monthly	Logistics indicator
Waterway freight volume	Monthly	Logistics indicator
Local financial revenue	Monthly	Policy indicator
Local financial expenditure share	Quarterly	Policy indicator
Power generation amount	Monthly	Power indicator
Power consumption amount	Monthly	Power indicator

Notes: (1) For the policy indicators, we use the ratio of local financial expenditure to GDP, referred to as the Local Financial Expenditure Share, to measure the degree of government intervention, following Li and Lin [21]. (2) Policy indicators are adjusted using a COVID-19 dummy variable to account for the effects of exceptional economic fluctuations during specific periods.

In the dynamic factor model, we assume that all indicators are influenced by a latent global factor *g*_*t*_. Moreover, indicators in each category *i*, i=1,⋯,6, are affected by a latent group-specific common factor *f*_*it*_. The GDP is influenced by all group-specific common factors $f_{1t},\cdots ,f_{6t}$. Following Ba$\overset{´}{\text{n}}$bura and Modugno [22], we also incorporate an individual-specific factor for each indicator to account for serial correlation in the residuals. Then, we assume that


$$y_{t}:=\left[\begin{array}{c} y_{t}^{Q}\\ y_{t}^{M} \end{array}\right]=\left[\begin{array}{ccccccc} C^{Q} & C^{Q} & C^{Q} & I_{m} & I_{m} & I_{m} & 0\\ C^{M} & 0 & 0 & 0 & 0 & 0 & I_{n} \end{array}\right]\left[\begin{array}{c} f_{t}\\ f_{t-1}\\ f_{t-2}\\ s_{t}^{Q}\\ s_{t-1}^{Q}\\ s_{t-2}^{Q}\\ s_{t}^{M} \end{array}\right]+\left[\begin{array}{c} e_{t}^{Q}\\ e_{t}^{M} \end{array}\right]$$

$$:=Cx_{t}+e_{t}$$ (1)

where $f_{t}=(g_{t},f_{1t},\cdots ,f_{6t}{)}^{T}$ is the vector consisting of the global factor and all group-specific common factors, $s_{t}^{Q}=(s_{1t}^{Q},\cdots ,s_{mt}^{Q}{)}^{T}$ and $s_{t}^{M}=(s_{1t}^{M},\cdots ,s_{nt}^{M}{)}^{T}$ are the vectors of individual-specific factors for monthly indicators and quarterly indicators, respectively, and $I_{n}$ denotes an n×n identity matrix. Here, $C^{Q}=\{C_{ij}^{Q}{\}}_{m\times 7}$ represents the coefficient matrix for quarterly indicators, where $C_{ij}^{Q}=0$ if indicator *i* is not affected by factor *j*. As suggested by Giannone *et al*. [10], we set the coefficient matrices of $f_{t-2}$, $f_{t-1}$ and $f_{t}$ to be identical for quarterly indicators because the impact of the factor over the three months to the quarterly indicator of the current quarter is assumed to be the same. Similarly, $C^{M}=\{C_{ij}^{Q}{\}}_{n\times 7}$ is the coefficient matrix for monthly indicators, where $C_{ij}^{M}=0$ if indicator *i* is not affected by factor *j*. Additionally, we assume that $e_{t}^{Q}\sim N\left(0,R^{Q}\right)$ and $e_{t}^{M}\sim N\left(0,R^{M}\right)$, where $R^{Q}$ and $R^{M}$ are diagonal matrices, and they are independent of each other. We further denote $y_{t}={\left((y_{t}^{Q}{)}^{T},(y_{t}^{M}{)}^{T}\right)}^{T}$, $x_{t}={\left(f_{t}^{T},f_{t-1}^{T},f_{t-2}^{T},(s_{t}^{Q}{)}^{T},(s_{t-1}^{Q}{)}^{T},(s_{t-2}^{Q}{)}^{T},(s_{t}^{M}{)}^{T}\right)}^{T}$ and $e_{t}={\left((e_{t}^{Q}{)}^{T},(e_{t}^{M}{)}^{T}\right)}^{T}$.

Note that the quarterly indicators $y_{t}^{Q}$ in Eq 1 are only observed when *t* = 3*l* for l=1,2,⋯. We use a matrix $I_{t}^{*}$ to extract the non-missing values of $y_{t}$ at time *t*. When $y_{t}$ contains no missing values, $I_{t}^{*}$ is the identity matrix. If some values of $y_{t}$ are missing, $I_{t}^{*}$ is the identity matrix with the corresponding rows removed. Define $y_{t}^{*}=I_{t}^{*}y_{t}$, $C_{t}^{*}=I_{t}^{*}C$, and $e_{t}^{*}=I_{t}^{*}e_{t}$. Then, the observation equation of the DFM at each time *t* can be written as

$$y_{t}^{*}=C_{t}^{*}x_{t}+e_{t}^{*},$$ (2)

where $e_{t}^{*}\sim N\left(0,R_{t}^{*}\right)$ with $R_{t}^{*}=I_{t}^{*}\left(\begin{array}{cc} R^{Q} & 0\\ 0 & R^{M} \end{array}\right)(I_{t}^{*}{)}^{T}$.

The state equation of the DFM is assumed to consist of AR(1) processes, which can be represented by


$$x_{t}:=\left[\begin{array}{c} f_{t}\\ f_{t-1}\\ f_{t-2}\\ s_{t}^{Q}\\ s_{t-1}^{Q}\\ s_{t-2}^{Q}\\ s_{t}^{M} \end{array}\right]=\left[\begin{array}{ccccccc} \gamma & 0 & 0 & 0 & 0 & 0 & 0\\ I_{7} & 0 & 0 & 0 & 0 & 0 & 0\\ 0 & I_{7} & 0 & 0 & 0 & 0 & 0\\ 0 & 0 & 0 & \alpha^{Q} & 0 & 0 & 0\\ 0 & 0 & 0 & I_{m} & 0 & 0 & 0\\ 0 & 0 & 0 & 0 & I_{m} & 0 & 0\\ 0 & 0 & 0 & 0 & 0 & 0 & \alpha^{M} \end{array}\right]\left[\begin{array}{c} f_{t-1}\\ f_{t-2}\\ f_{t-3}\\ s_{t-1}^{Q}\\ s_{t-2}^{Q}\\ s_{t-3}^{Q}\\ s_{t-1}^{M} \end{array}\right]+\left[\begin{array}{c} u_{t}\\ 0\\ 0\\ a_{t}^{Q}\\ 0\\ 0\\ a_{t}^{M} \end{array}\right]$$

$$:=Ax_{t-1}+a_{t},$$ (3)

where $\gamma =diag\left(\gamma_{g},\gamma_{f,1},\cdots ,\gamma_{f,6}\right)$, $\alpha^{Q}=diag\left(\alpha_{1}^{Q},\ldots ,\alpha_{m}^{Q}\right)$ and $\alpha^{M}=diag\left(\alpha_{1}^{M},\ldots ,\alpha_{n}^{M}\right)$. We also assume that $u_{t}\sim N\left(0,\Sigma_{u}\right)$, $a_{t}^{Q}\sim N\left(0,\Sigma_{a}^{Q}\right)$ and $a_{t}^{M}\sim N\left(0,\Sigma_{a}^{M}\right)$ , where $\Sigma_{u}$, $\Sigma_{a}^{Q}$ and $\Sigma_{a}^{M}$ are diagonal matrices, and they are independent of each other.

The dynamic factor model defined by Eqs 2 and (3) is a linear-Gaussian state space model. We can estimate the model parameters $\theta =(\gamma ,\alpha^{Q},\alpha^{M}$, $\Sigma_{u},\Sigma_{a}^{Q},\Sigma_{a}^{M}$, $C^{Q},R^{Q},C^{M},R^{M})$ using the Expectation-Maximization (EM) algorithm as in Giannone *et al*. [10] and Ba$\overset{´}{\text{n}}$bura and Modugno [22]. The detailed algorithm is presented in S1 Appendix.

Now, we turn our attention to nowcasting of the year-on-year growth rate of quarterly GDP. Specifically, at times t=3l−2, t=3l−1, and *t* = 3*l*, we consider predicting $y_{1,3l}^{Q}$ using the DFM, denoted by ${\hat{y}}_{1,3l|t}^{Q,DFM}$. It is important to note that quarterly GDP data is released with a delay of more than half a month, we still need to predict $y_{1,3l}^{Q}$ at time *t* = 3*l*. At each time t=3l−2, t=3l−1, and *t* = 3*l*, we first estimate the model parameters based on the data available up to time 3(*l*–1). Then, we have


$${\hat{y}}_{1,3l|3l-2}^{Q,DFM}=E(y_{1,3l}^{Q}\;|\;y_{1}^{*},\cdots ,y_{3l-2}^{*};\hat{\theta }),$$

$${\hat{y}}_{1,3l|3l-1}^{Q,DFM}=E(y_{1,3l}^{Q}\;|\;y_{1}^{*},\cdots ,y_{3l-1}^{*};\hat{\theta }),$$

and


$${\hat{y}}_{1,3l|3l}^{Q,DFM}=E(y_{1,3l}^{Q}\;|\;y_{1}^{*},\cdots ,y_{3l-1}^{*},y_{3l}^{M},y_{3l,2}^{Q},\cdots ,y_{3l,m}^{Q};\hat{\theta }).$$

for *t* = 3*l*−2, *t* = 3*l*−1, and *t* = 3*l*, respectively. Here, $E(y_{1,3l}^{Q}\;|\;y_{1}^{*},\cdots ,y_{3l-2}^{*};\hat{\theta })$, $E(y_{1,3l}^{Q}\;|\;y_{1}^{*},\cdots ,y_{3l-1}^{*};\hat{\theta })$ and $E(y_{1,3l}^{Q}\;|\;y_{1}^{*},\cdots ,$ $y_{3l-1}^{*},y_{3l}^{M},y_{3l,2}^{Q},\cdots ,y_{3l,m}^{Q};\hat{\theta })$ can be obtained through the Kalman filter as in S2 Appendix.

### 2.2 Regression model using electricity data

**Fig 1:**
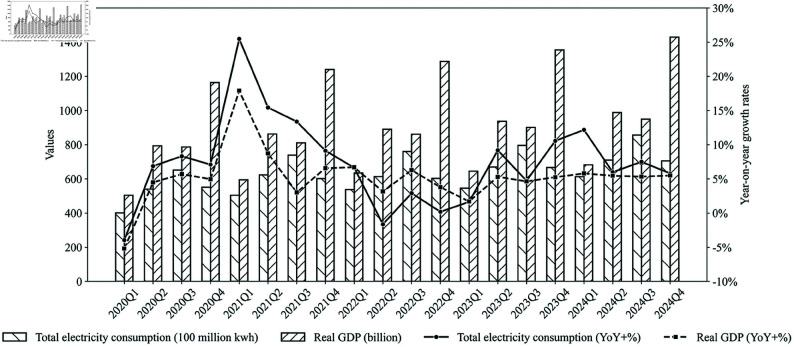
Quarterly electricity consumption and GDP in Fujian Province, China.

Many studies have identified a strong correlation between electricity usage and GDP [16–18]. Fig 1 reports the quarterly electricity consumption and actual GDP in Fujian Province for each quarter from the first quarter of 2020 to the third quarter of 2024, along with their respective growth rates. Although economic growth outpaced the increase in electricity demand during 2022 due to a notable economic recovery, the long-term trend shows that the growth rates of electricity consumption and economic growth generally move in the same direction. This relationship allows us to use real-time electricity consumption data for GDP nowcasting.

When forecasting the quarterly GDP during the first two months of a quarter, the electricity consumption data for the entire quarter is not yet available. The bridge model proposed by Parigi and Schlitzer [1] suggested using predicted values of the unpublished data instead of their true values to forecast GDP. However, multi-step predictions tend to amplify the prediction error.

Here, we introduce the net increase of electricity capacity as a new predictor variable, which reflects the expectations for electricity demand in the subsequent months, containing important information for electricity consumption forecasts, as shown in Zhang *et al*. [23]. Specifically, to predict $y_{1,3l}^{Q}$, the year-on-year GDP growth rate in quarter *l*, at times t=3l−2, *t* = 3*l*−1, and *t* = 3*l*, we use the following regression models

$$y_{1,3l}^{Q}=\beta_{10}+\beta_{11}\;y_{1,3(l-1)}^{Q}+\beta_{12}\;r_{E,3l-2}+\beta_{13}\;r_{I,3l-2}+\varepsilon_{1},$$ (4)

$$y_{1,3l}^{Q}=\beta_{20}+\beta_{21}\;y_{1,3(l-1)}^{Q}+\beta_{22}\;r_{E,3l-2}+\beta_{23}\;r_{E,3l-1}+\beta_{24}\;r_{I,3l-1}+\varepsilon_{2},$$ (5)

and

$$y_{1,3l}^{Q}=\beta_{30}+\beta_{31}\;y_{1,3(l-1)}^{Q}+\beta_{32}\;r_{E,3l-2}+\beta_{33}\;r_{E,3l-1}+\beta_{34}\;r_{E,3l}+\varepsilon_{3},$$ (6)

respectively. Let *E*_*t*_ be the electricity consumption in month *t*, and let *I*_*t*_ be the net increase of electricity capacity in the past three months. In the above models Eqs 4, (5), and (6),


$$r_{E,t}=\frac{E_{t}-E_{t-12}}{E_{t-12}}$$

represents the year-on-year electricity consumption growth rate in month *t*, and


$$r_{I,t}=\frac{I_{t}-I_{t-12}}{E_{t-12}}$$

represents the rate of change in the last three months’ net increase of electricity capacity over one year relative to the electricity consumption of the same month in the previous year.

In empirical studies, it is found that the responsiveness of changes in economic growth to changes in electricity consumption is related to various factors, such as time cycles, industrial structure, and technological progress. For example, Lee and Chang [24] found that changes in industrial structure affect the quantitative relationship between economic growth and electricity consumption through Hansen’s test. Yuan *et al*. [25] discovered that the relationship between electricity consumption and economic growth in China exhibits cyclicality. Therefore, to ensure that the model parameters accurately capture the current quantitative relationship between electricity consumption and economic development, we employ a rolling window approach for GDP forecasting. Considering that the industrial structure and technological development are relatively stable within a 2 to 3-year period, as demonstrated in Malerba and Orsenigo [26] and Foster *et al*. [27], we have set the rolling window to 9 quarters. We denote the forecasts of $y_{1,3l}^{Q}$ by models (4), (5), and (6) as ${\hat{y}}_{1,3l|3l-2}^{Q,REG}$, ${\hat{y}}_{1,3l|3l-1}^{Q,REG}$, and ${\hat{y}}_{1,3l|3l}^{Q,REG}$, respectively.

### 2.3 Model averaging

The DFM captures the long-term relationship between GDP and other economic indicators through common factors, utilizing this relationship for GDP forecasting. Meanwhile, the regression models uses electricity data, which are closely related to production, to capture short-term economic changes and predict GDP growth rates. To improve forecast accuracy and stability, we propose using model averaging to combine predictions from the DFM and the regression models.

We use a linear combination approach for model averaging, in which the composite prediction is a weighted average of the predictions from both the DFM and the regression models. Following Dong *et al*. [28], the weights assigned to each model are determined based on the inverse of the predicted mean absolute error (MAE) or root mean squared error (RMSE). Recall that the predicted values of the year-on-year GDP growth rate by the DFM and the regression models are ${\hat{y}}_{1,3l|3l-k}^{Q,DFM}$ and ${\hat{y}}_{1,3l|3l-k}^{Q,REG}$, k=0,1,2, respectively. The prediction errors associated with the DFM and regression models are denoted as $e_{3l|3l-k}^{DFM}=y_{1,3l}^{Q}\;-\;{\hat{y}}_{1,3l|3l-k}^{Q,DFM}$ and $e_{3l|3l-k}^{REG}=y_{1,3l}^{Q}\;-\;{\hat{y}}_{1,3l|3l\;-\;k}^{Q,REG},$ respectively. Assume that we want to predict $y_{1,3L}^{Q}$, the year-on-year GDP growth rate of quarter *L*. Then, the composite prediction for $y_{1,3L}^{Q}$ at time t=3L−k for k=0,1,2 is


$${\hat{y}}_{1,3L|3L-k}^{Q,COM}=w_{3L|3L-k}^{DFM}\times {\hat{y}}_{1,3L|3L-k}^{Q,DFM}+w_{3L|3L-k}^{REG}\times {\hat{y}}_{1,3L|3L-k}^{Q,REG},$$

where the weight of the DFM is

$$w_{3L|3L-k}^{DFM}=w_{3L|3L-k}^{*DFM}/(w_{3L|3L-k}^{*DFM}+w_{3L|3L-k}^{*REG}),$$ (7)

and the weight of the regression model is

$$w_{3L|3L-k}^{REG}=w_{3L|3L-k}^{*REG}/(w_{3L|3L-k}^{*DFM}+w_{3L|3L-k}^{*REG}).$$ (8)

Here, we let $w_{3L|3L-k}^{*DFM}={\left(\sum_{l=1}^{L-1}|e_{3l|3l-k}^{DFM}|\right)}^{-1}$ and $w_{3L|3L-k}^{*REG}={\left(\sum_{l=1}^{L-1}|e_{3l|3l-k}^{REG}|\right)}^{-1}$ when the MAE is used as the performance measurement metric, and let $w_{3L|3L-k}^{*DFM}={\left[\sum_{l=1}^{L-1}(e_{3l|3l-k}^{DFM}{)}^{2}\right]}^{-1/2}$ and $w_{3L|3L-k}^{*REG}={\left[\sum_{l=1}^{L-1}(e_{3l|3l-k}^{REG}{)}^{2}\right]}^{-1/2}$ when the RMSE is used as the performance measurement metric.

## 3 Empirical results

In this section, we use data from Fujian province, China, to demonstrate the effectiveness of our proposed GDP nowcasting models. We collected GDP data and other macroeconomic indicators for the period from January 2010 to December 2024 from multiple sources, including the Wind database, the Fujian Provincial Bureau of Statistics, and the Fujian Provincial Ministry of Finance. Additionally, we obtained monthly electricity consumption data and monthly changes in electricity capacity for the period from January 2019 to December 2024 from the State Grid Fujian Electric Power Co., Ltd., Fujian, China.

The Spring Festival, one of China’s most important holidays, significantly impacts China’s economic activities. Therefore, we need to eliminate the impact of the Spring Festival effect before forecasting GDP. It’s important to note that the Spring Festival is determined by the lunar calendar and typically falls in January or February. Following Shu and Tsang [29], we aggregate the monthly data for the first two months of each year and allocate them to January and February based on the proportion of days to eliminate the impact of the Spring Festival effect. Then, we convert the data into year-on-year growth rates to further eliminate the effects of other holidays, temperature, seasonal variations, and cyclical factors.

When conducting nowcasting, it is noted that similar to GDP, the release of some economic indicators is subject to significant delays, including value added of industries above designated size, operating revenue industrial enterprise, per capita disposable income of urban residents, total retail sales of consumer goods, fixed asset investment, local financial revenue, and local financial expenditure share. Following Jansen *et al*. [30], we adopt a pseudo real-time design. That is, we use unadjusted data, and when conducting GDP nowcasting at the end of each month for the current quarter, we only use the data that have been released by the end of that month.

### 3.1 Results of DFM using macroeconomic indicators

**Fig 2:**
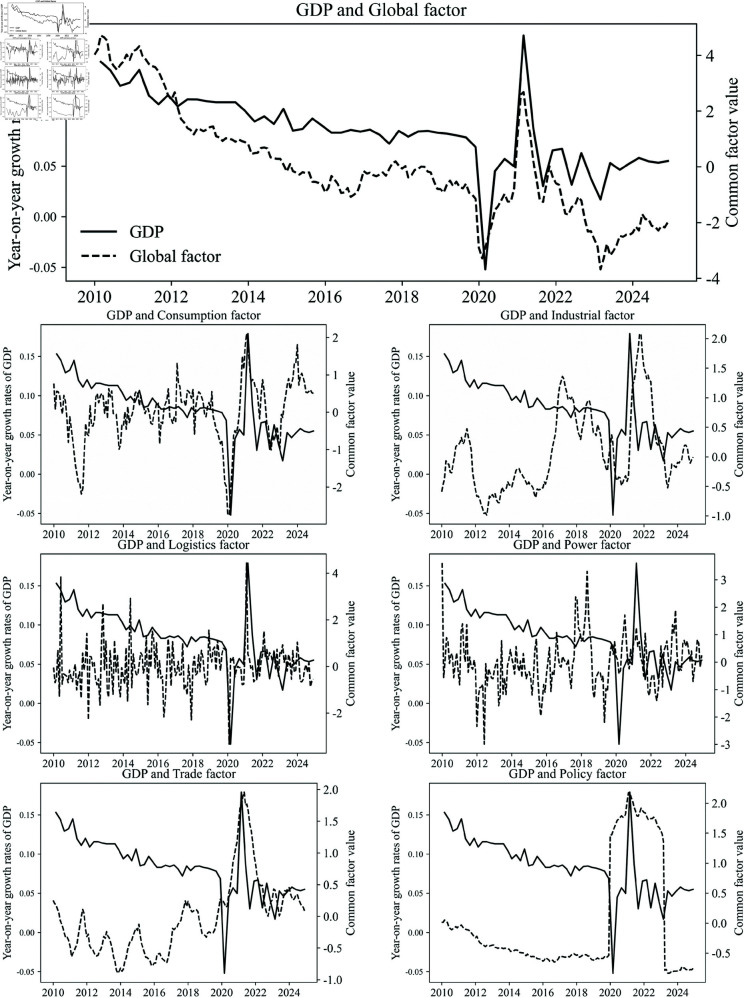
Estimated global factor and group-specific common factors (dashed lines), along with the true GDP growth rates (solid lines).

We first estimate the DFM using GDP and other macroeconomic indicators over the entire period from January 2010 to December 2024. The estimated global factor and group-specific common factors are plotted in Fig 2, along with the actual year-on-year GDP growth rate. It can be seen that the global factor, which comprehensively integrates various indicators, exhibits a strong correlation with the GDP growth rate. Meanwhile, the group-specific common factors are capable to capture the trends and directions of economic growth during specific periods.

**Fig 3:**
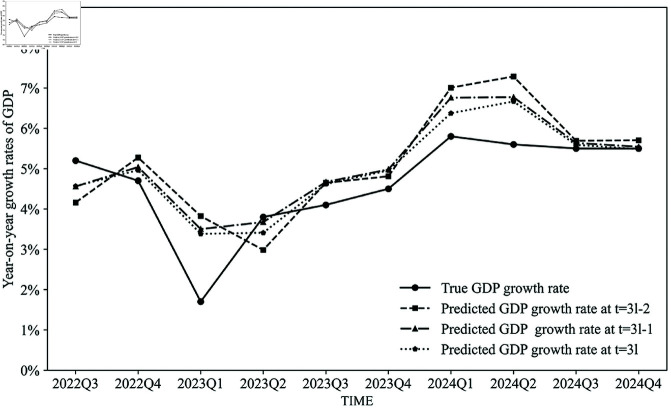
Prediction results of the DFM.

Next, we apply the DFM to conduct nowcasting for the quarterly GDP growth rate from the third quarter of 2022 to the fourth quarter of 2024. When predicting the year-on-year GDP growth rate in quarter *l* at times t=3l−2, t=3l−1, and *t* = 3*l*, we utilize all data released from January 2010 up to the end of month *t* for the prediction. Fig 3 presents the predicted quarterly GDP growth rates at quarter *l* in the first month (t=3l−2), the second month (t=3l−1), and the third month (*t* = 3*l*) of that quarter. As observed from Fig 3, the prediction results obtained in the third month of the quarter are more accurate than those obtained in the first or two months, as we have more monthly data available to use.

### 3.2 Regression results using electricity data

Now, we consider using the regression models (4), (5), and (6) to predict the year-on-year GDP growth rate in quarter *l* at times t=3l−2, t=3l−1, and *t* = 3*l*, respectively. Similarly, we conduct nowcasting for the quarterly GDP growth rates from the third quarter of 2022 to the fourth quarter of 2024. Due to data limitations, we use electricity data from January 2020 to month *t* for the prediction.

In the first month and the second month of each quarter, we compare the prediction performance of our proposed model with the bridge model proposed by Parigi and Schlitzer [1]. Particularly, the bridge model assumes


$$y_{1,3l}^{Q}=\beta_{10}+\beta_{11}\;y_{1,3(l-1)}^{Q}+\beta_{12}\;r_{E,3l-2}+\beta_{13}\;{\hat{r}}_{E,3l-1}+\beta_{14}\;{\hat{r}}_{E,3l}+\varepsilon_{1},$$

and


$$y_{1,3l}^{Q}=\beta_{20}+\beta_{21}\;y_{1,3(l-1)}^{Q}+\beta_{22}\;r_{E,3l-2}+\beta_{23}\;r_{E,3l-1}+\beta_{24}\;{\hat{r}}_{E,3l}+\varepsilon_{2},$$

for t=3l−2 and t=3l−1, respectively. Here, ${\hat{r}}_{E,3l\;-\;1}$ and ${\hat{r}}_{E,3l}$ represent the predicted values for $r_{E,3l\;-\;1}$ and *r*_*E*,3*l*_, using the AR(1) model based on the data available up to time *t*. Table 2 compares the predicted results using the bridge model and our proposed regression models from the third quarter of 2022 to the fourth quarter of 2024. We use the MAE and RMSE as the metrics for prediction accuracy, which are, respectively,

**Table 2:** MAEs and RMSEs for the bridge model and our proposed regression models.

Error Metric	Model	The First Month	The Second Month
MAE	Regression Model	0.690%	0.669%
	Bridge Model	1.058%	0.923%
RMSE	Regression Model	0.981%	0.954%
	Bridge Model	2.005%	1.499%


$${MAE}_{k}=\frac{1}{L}\sum_{l=1}^{L}\left|y_{1,3l|3l-k}^{Q,true}-{\hat{y}}_{1,3l|3l-k}^{Q,pred}\right|,k=0,1,2.$$

and


$${RMSE}_{k}=\sqrt{\frac{1}{L}\sum_{l=1}^{L}{\left(y_{1,3l|3l-k}^{Q,true}-{\hat{y}}_{1,3l|3l-k}^{Q,pred}\right)}^{2}},\;k=0,1,2.$$

As shown in Table 2, the MAEs and RMSEs of our proposed regression models, which include changes in electricity capacity, are smaller than that of the bridge model.

**Fig 4:**
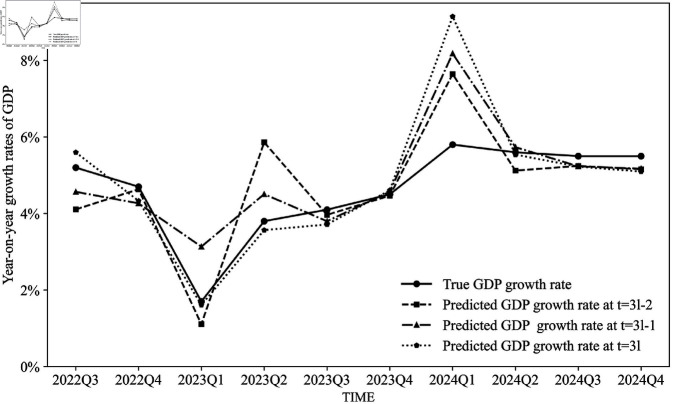
Prediction results of the regression models.

Fig 4 plots the predicted GDP growth rates using our proposed regression models at quarter *l* in the first month (*t* = 3*l*−2), the second month (*t* = 3*l*−1), and the third month (*t* = 3*l*) of that quarter, along with the actual GDP growth rates. We find that the regression models exhibit high sensitivity to fluctuations in economic growth. Furthermore, the closer the prediction time is to the official GDP disclosure date, the more precise the model’s predictions become.

### 3.3 Results of model averaging

Finally, we use the model averaging approach to combine predictions from the DFM and the regression models. Again, the out-of-sample prediction period is from the third quarter of 2022 to the fourth quarter of 2024. To make the combination weights more stable, we set the weights of the DFM and the regression models to be equal from the third quarter of 2022 to the first quarter of 2023. Then, from the second quarter of 2023 to the fourth quarter of 2024, the weights of the DFM and the regression models are calculated using Eq 7 and Eq 8, respectively.

**Fig 5:**
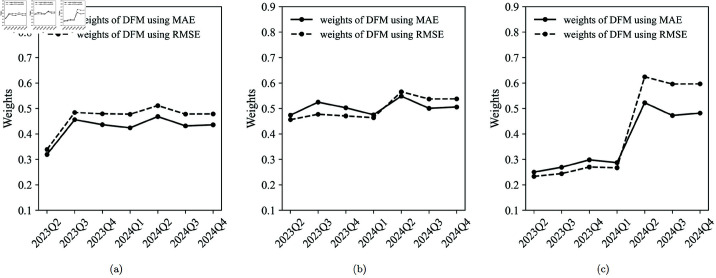
Weights of the DFM. (a) Weights in the first month of the quarter (*t*=3*l*-2). (b) Weights in the second month of the quarter (*t*=3*l*-1). (c) Weights in the third month of the quarter (*t*=3*l*).

Fig 5 reports the weights of the DFM and the regression models at quarter *l* in the first month (t=3l−2), the second month (t=3l−1), and the third month (*t* = 3*l*) of that quarter from the second quarter of 2023 to the fourth quarter of 2024.

**Fig 6:**
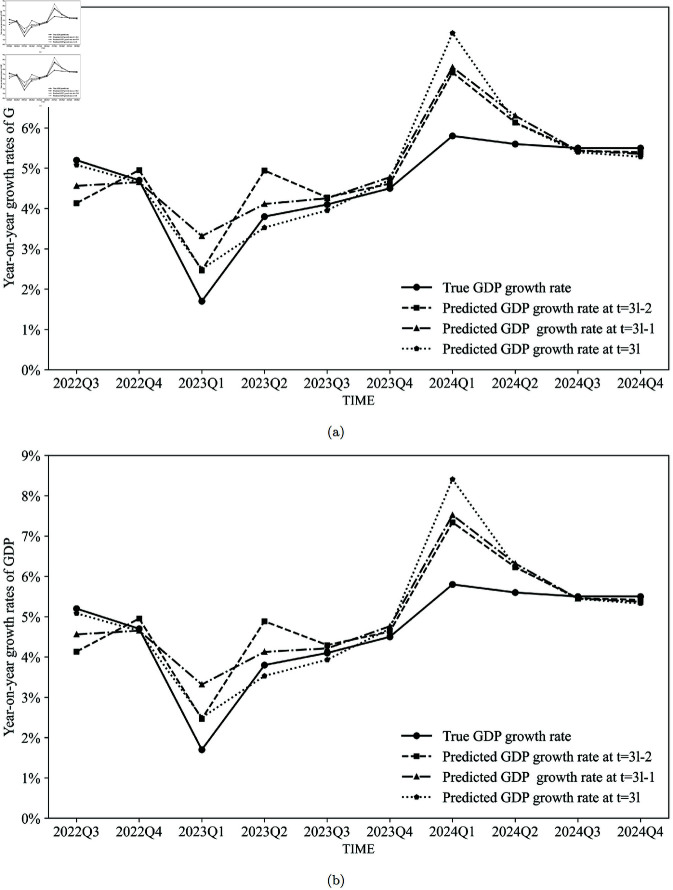
Prediction results of the composite model. (a) The composite model weighted using MAE. (b) The composite model weighted using RMSE.

Furthermore, the nowcasting results of the composite model for quarter *l* in the first month (*t* = 3*l*−2), the second month (*t* = 3*l*−1), and the third month (*t* = 3*l*) of that quarter are shown in Fig 6. As more information becomes available, the accuracy of nowcasting improves progressively.

Table 3 presents the nowcasting MAEs and RMSEs of the DFM, the regression model, and the composite model, respectively. Again, it demonstrates that as the prediction time approaches the GDP disclosure date, the MAEs of the predictions decrease. Additionally, the composite model enhances the prediction accuracy of the year-on-year quarterly GDP growth rate, measured by MAEs, compared to both the DFM and the regression models. However, the performances of RMSEs are suboptimal, since it is sensitive to outliers. Due to the adjustment of COVID-19 prevention and control policies in China, the economic operation of Chinese society was significantly impacted in the first quarter of 2023, with both GDP growth rates and electricity consumption showing marked declines. As illustrated in Fig 3, the nowcasting results for the first quarter of 2023 were considerably overestimated by the DFM due to its inherent inertia in predictions. In contrast, the regression model based on electricity data exhibited a substantial overestimation in its nowcasting results for the first quarter of 2024. This overestimation can be attributed to the low base level of electricity consumption in the first quarter of 2023, which led to an abnormally high year-on-year growth rate in electricity consumption for the first quarter of 2024.

**Table 3:** Nowcasting MAEs and RMSEs of various models from 2022Q3 to 2024Q4.

Error Metric	Prediction Models	The First Month	The Second Month	The Third Month
MAE	Dynamic Factor Model	0.870%	0.626%	0.569%
	Regression Model	0.690%	0.669%	0.567%
	Composite Model	0.580%	0.566%	0.499%
RMSE	Dynamic Factor Model	1.066%	0.815%	0.735%
	Regression Model	0.981%	0.954%	1.094%
	Composite Model	0.762%	0.819%	0.897%

Therefore, we may consider the first quarters of 2023 and 2024 as outliers and exclude these two quarters from the evaluation metrics. Table 4 reports the nowcasting MAEs and RMSEs of the DFM, the regression model, and the composite model, respectively, with the results for 2023Q1 and 2024Q1 excluded. The composite model is found to exhibit more accurate predictions than both the DFM and the regression model, as measured by both MAE and RMSE. Specifically, in the third month of a quarter (*t* = 3*l*), the composite model’s prediction MAE and RMSE are, respectively, 54.3% and 56.0% lower than those of the dynamic factor model, and 29.7% and 24.1% lower than those of the regression model.

**Table 4:** Nowcasting MAEs and RMSEs of various models excluding 2023Q1 and 2024Q1.

Error Metric	Prediction Models	The First Month	The Second Month	The Third Month
MAE	Dynamic Factor Model	0.671%	0.438%	0.429%
	Regression Model	0.558%	0.359%	0.279%
	Composite Model	0.391%	0.304%	0.196%
RMSE	Dynamic Factor Model	0.821%	0.558%	0.529%
	Regression Model	0.857%	0.416%	0.307%
	Composite Model	0.544%	0.371%	0.233%

Finally, we conducted the Diebold-Mariano test [31] to assess the significance of the improvement in forecast accuracy of the composite model compared to the DFM and the regression model. The corresponding *p*-values are reported in Table 5. The results indicate that, the composite model’s absolute prediction error is reduced compared to both the DFM and regression model at the 10% significance level. And its squared prediction error has a significant reduction compared to the regression model at the 5% level. What’s more, after excluding the outliers in 2023Q1 and 2024Q1, the decrease in the composite model’s absolute prediction error compared to the regression model is statistically significant at the 10% level. Additionally, the composite model exhibits a statistically significant reduction in both absolute error and squared error compared to the DFM at the 1% level.

**Table 5:** *p*-values of the Diebold-Mariano test.

Error Metric	Panel A: All out-of-sample		Panel B: Partial out-of-sample (except Q1)	
	REG vs. COM	DFM vs. COM	REG vs. COM	DFM vs. COM
Absolute Error	0.076	0.094	0.089	0.000
Squared Error	0.046	0.373	0.149	0.005

## 4 Conclusion

This paper introduces a composite GDP nowcasting model using macroeconomic indicators and electricity data. We first consider two distinct GDP nowcasting models. One is the DFM, which utilizes the linkage between GDP and other macroeconomic indicators. Additionally, the DFM is capable of handling mixed-frequency data. The other one is the regression model using electricity data, which leverages the cointegration relationship between electricity consumption and economic growth. To address the issue that the total electricity consumption for the entire quarter is not observable when forecasting the quarterly GDP during the first two months of that quarter, we introduce changes in electricity capacity into the regression model to reflect expectations for future electricity demand. Then, model averaging is employed to combine predictions from the DFM and the regression model. Finally, we use macroeconomic indicators and electricity data from Fujian Province, China, to forecast its year-on-year quarterly GDP growth rate. The prediction results demonstrate the effectiveness of our proposed model.

Notice that electricity consumption is greatly affected by temperature. When using electricity data to predict GDP, although year-on-year data can eliminate the seasonal changes in temperature, temperature fluctuations still have a significant impact on the forecast results. Effectively removing the impact of temperature can be warranted as a future research topic. What’s more, although certain adjustments help the model maintain stability, unexpected events and partial delay of data may still introduce considerable uncertainties into the GDP nowcasting. Strengthening the model’s robustness for predictions during these periods could be a valuable direction for our future optimization.

## Supporting information

S1 Appendix Parameter estimation for DFM (PDF)

S2 Appendix Prediction in DFM. (PDF)

S1 File Available data used in this article. (XLS)
