# Supplementary material for: Composite GDP nowcasting using macroeconomic variables and electricity data
Source: PLoS One. 2025 Jun 9;20(6):e0324381. doi: 10.1371/journal.pone.0324381 (PMC12148135; doi:10.1371/journal.pone.0324381)
Supplement: S1 Appendix — (PDF) [file pone.0324381.s001.pdf]

S1 Appendix. Parameter Estimation for DFM.

In this section, we introduce how to estimate the parameters $\boldsymbol{\theta} = \Big(\boldsymbol{\gamma}, \boldsymbol{\alpha}^Q, \boldsymbol{\alpha}^M, \Sigma_u, \Sigma_a^Q, \Sigma_a^M, \boldsymbol{C}^Q, \boldsymbol{R}^Q, \boldsymbol{C}^M, \boldsymbol{R}^M\Big)$ in the DFM proposed in Section 2.1 using all available indicators up to time t.

The parameters in the proposed DFM can be estimated using the Expectation-Maximization (EM) algorithm. Following Giannone et al. [10] and Bańbura and Modugno [23], we apply the principal component method to set the initial values of the model parameters. Particularly, suppose that $y_{k,1,s}, \cdots, y_{k,n_k,s}$, $s = 1, \cdots, t$, are all indicators influenced by the k-th factor, we set the initial value of the k-th factor as the first principal component of $y_{k,1,s}, \cdots, y_{k,n_k,s}$. Once we have obtained the initial values of the latent factors, the initial values of the model parameters can be set through the least square method. Then, we iteratively proceed with the following E-step and M-step to respectively update the latent states and model parameters until the algorithm converges.

E-step: Suppose that we already have an estimate $\widehat{\boldsymbol{\theta}} = \Big(\widehat{\boldsymbol{\gamma}}, \widehat{\boldsymbol{\alpha}}^Q, \widehat{\boldsymbol{\alpha}}^M, \widehat{\Sigma}_u, \widehat{\Sigma}_a^Q, \widehat{\Sigma}_a^M, \widehat{\boldsymbol{C}}^Q, \widehat{\boldsymbol{R}}^Q, \widehat{\boldsymbol{C}}^M, \widehat{\boldsymbol{R}}^M\Big)$ of the parameters. Let $\widehat{\boldsymbol{C}}_s^*$, $\widehat{\boldsymbol{R}}_s^*$, $\widehat{\boldsymbol{A}}$, and $\widehat{\boldsymbol{Q}}$ be the corresponding estimates of $\boldsymbol{C}_s^*$, $\boldsymbol{R}_s^*$, $\boldsymbol{A}$, and $\boldsymbol{Q}$ defined in (2) and (3), respectively. Here $\boldsymbol{Q}$ is the covariance matrix of $\boldsymbol{a}_t$ defined in (3). We calculate

$$\boldsymbol{x}_{s|t} := E\big(\boldsymbol{x}_s \,|\, \boldsymbol{y}_1^*, \cdots, \boldsymbol{y}_t^*; \widehat{\boldsymbol{\theta}}\big), \quad \boldsymbol{V}_{s|t} := \mathrm{Var}\big(\boldsymbol{x}_s \,|\, \boldsymbol{y}_1^*, \cdots, \boldsymbol{y}_t^*; \widehat{\boldsymbol{\theta}}\big),$$

and

$$\boldsymbol{V}_{s|t}^- = \mathrm{Cov}\big(\boldsymbol{x}_{s-1}, \boldsymbol{x}_s \,|\, \boldsymbol{y}_1^*, \cdots, \boldsymbol{y}_t^*; \widehat{\boldsymbol{\theta}}\big),$$

for $s = 1, 2, \cdots, t$ using the Kalman filter as follows.

Step 1. Let $\boldsymbol{x}_{0|0}$ and $\boldsymbol{V}_{0|0}$ be the zero vector and zero matrix correspondingly. For $s = 1, \cdots, t$, calculate

$$\boldsymbol{x}_{s|s-1} = \widehat{\boldsymbol{A}}\boldsymbol{x}_{s-1|s-1}, \quad \boldsymbol{V}_{s|s-1} = \widehat{\boldsymbol{A}}\boldsymbol{V}_{s-1|s-1}\widehat{\boldsymbol{A}}^T + \widehat{\boldsymbol{Q}},$$

and

$$\boldsymbol{x}_{s|s} = \boldsymbol{x}_{s|s-1} + \boldsymbol{K}_s\left(\boldsymbol{y}_s^* - \widehat{\boldsymbol{C}}_s^*\boldsymbol{x}_{s|s-1}\right), \quad \boldsymbol{V}_{s|s} = \left(\boldsymbol{I}_p - \boldsymbol{K}_s\widehat{\boldsymbol{C}}_s^*\right)\boldsymbol{V}_{s|s-1},$$

where $\boldsymbol{K}_s = \boldsymbol{V}_{s|s-1}\big(\widehat{\boldsymbol{C}}_s^*\big)^T\left(\widehat{\boldsymbol{C}}_s^*\boldsymbol{V}_{s|s-1}\big(\widehat{\boldsymbol{C}}_s^*\big)^T + \widehat{\boldsymbol{R}}_s^*\right)^{-1}$ is the so-called “Kalman gain”, and p is the dimension of $\boldsymbol{x}_s$.

Step 2. After obtained $\boldsymbol{x}_{t|t}$ and $\boldsymbol{V}_{t|t}$ in the above step, let $\boldsymbol{V}_{t|t}^- = \left(\boldsymbol{I}_p - \boldsymbol{K}_t\widehat{\boldsymbol{C}}_t\right)\widehat{\boldsymbol{A}}\boldsymbol{V}_{t-1|t-1}$. Calculate $\boldsymbol{x}_{s-1|t}$ and $\boldsymbol{V}_{s-1|t}$ backward from $s = t$ to $s = 2$ by letting

$$\boldsymbol{x}_{s-1|t} = \boldsymbol{x}_{s-1|s-1} + \boldsymbol{G}_{s-1}\left(\boldsymbol{x}_{s|t} - \boldsymbol{x}_{s|s-1}\right),$$

$$\boldsymbol{V}_{s-1|t} = \boldsymbol{V}_{s-1|s-1} + \boldsymbol{G}_{s-1}\left(\boldsymbol{V}_{s|t} - \boldsymbol{V}_{s|s-1}\right)\boldsymbol{G}_{s-1}{}^T,$$

and

$$\boldsymbol{V}_{s-1|t}^- = \boldsymbol{V}_{s-1|s-1}\boldsymbol{G}_{s-2}{}^T + \boldsymbol{G}_{s-1}\left(\boldsymbol{V}_{s|t}^- - \widehat{\boldsymbol{A}}\boldsymbol{V}_{s-1|s-1}\right)\boldsymbol{G}_{s-2}{}^T,$$

where $\boldsymbol{G}_{s-1} = \boldsymbol{V}_{s-1|s-1}\widehat{\boldsymbol{A}}^T\boldsymbol{V}_{s|s-1}^{-1}$.

M-step: Suppose we already have $\boldsymbol{x}_{s|t}$, $\boldsymbol{V}_{s|t}$, and $\boldsymbol{V}^{-}_{s|t}$ for $s = 0, 1, \cdots, t$. According to the definition of $\boldsymbol{x}_t$, we denote $\boldsymbol{x}_{s|t}$ as

$$\boldsymbol{x}_{s|t} := \Big((\boldsymbol{f}_{s|t})^T, (\boldsymbol{f}_{s-1|t})^T, (\boldsymbol{f}_{s-2|t})^T, (\boldsymbol{s}^Q_{s|t})^T, (\boldsymbol{s}^Q_{s-1|t})^T, (\boldsymbol{s}^Q_{s-2|t})^T, (\boldsymbol{s}^M_{s|t})^T \Big)^T .$$

The model parameters are updated as follows.

Step 1. Update $\boldsymbol{\gamma}$, Σ_u, $\boldsymbol{\alpha}^Q$, $\boldsymbol{\alpha}^M$, Σ^Q_a, and Σ^M_a: Recall that g_s is the global factor and is the first dimension of $\boldsymbol{f}_s$. The state equation in Eq (3) indicates that

$$g_s = \gamma_g g_{s-1} + u_{1t},$$

where $u_{1t} \sim N(0, \Sigma_{u,11})$ with $\Sigma_{u,ij}$ being the (i,j)-th entry in the matrix Σ_u. We update γ_g and $\Sigma_{u,11}$ by letting

$$\widehat{\gamma}_g = \Omega^{-1}\left(g_{0:t-1}, g_{0:t-1}\right) \Omega\left(g_{0:t-1}, g_{1:t}\right)$$

and

$$\widehat{\Sigma}_{u,11} = \frac{1}{t}\left[\Omega\left(g_{1:t}, g_{1:t}\right) - \Omega\left(g_{0:t-1}, g_{1:t}\right)\widehat{\gamma}_g\right],$$

where

$$\Omega\left(g_{0:t-1}, g_{0:t-1}\right) = \sum_{s=1}^{t} \Big[\boldsymbol{f}_{s-1|t,1} \times \boldsymbol{f}_{s-1|t,1} + \mathrm{Var}\big(g_{s-1} \,|\, \boldsymbol{y}^*_1, \cdots, \boldsymbol{y}^*_t; \widehat{\boldsymbol{\theta}}\big) \Big],$$

$$\Omega\left(g_{0:t-1}, g_{1:t}\right) = \sum_{s=1}^{t} \Big[\boldsymbol{f}_{s-1|t,1} \times \boldsymbol{f}_{s|t,1} + \mathrm{Cov}\big(g_s, g_{s-1} \,|\, \boldsymbol{y}^*_1, \cdots, \boldsymbol{y}^*_t; \widehat{\boldsymbol{\theta}}\big) \Big],$$

and

$$\Omega\left(g_{1:t}, g_{1:t}\right) = \sum_{s=1}^{t} \Big[\boldsymbol{f}_{s|t,1} \times \boldsymbol{f}_{s|t,1} + \mathrm{Var}\big(g_s \,|\, \boldsymbol{y}^*_1, \cdots, \boldsymbol{y}^*_t; \widehat{\boldsymbol{\theta}}\big) \Big].$$

Here, $\boldsymbol{f}_{s|t,i}$ denotes the i-th dimension of the vector $\boldsymbol{f}_{s|t}$, and $\mathrm{Var}\big(g_{s-1} \,|\, \boldsymbol{y}^*_1, \cdots, \boldsymbol{y}^*_t; \widehat{\boldsymbol{\theta}}\big)$ and $\mathrm{Cov}\big(g_s, g_{s-1} \,|\, \boldsymbol{y}^*_1, \cdots, \boldsymbol{y}^*_t; \widehat{\boldsymbol{\theta}}\big)$ are given in $\boldsymbol{V}_{s|t}$ and $\boldsymbol{V}^{-}_{s|t}$, respectively. Other parameters in $\boldsymbol{\gamma}$, Σ_u, $\boldsymbol{\alpha}^Q$, $\boldsymbol{\alpha}^M$, Σ^Q_a, and Σ^M_a can be updated in a similar way.

Step 2. Update $\boldsymbol{C}^M$ and $\boldsymbol{R}^M$: Let $\boldsymbol{x}^M_{s|t} := \Big((\boldsymbol{f}_{s|t})^T, (\boldsymbol{s}^M_{s|t})^T \Big)^T$ be the sub-vector of $\boldsymbol{x}_{s|t}$ that is related to the monthly variables. We update the $\boldsymbol{C}^M$ by letting

$$\mathrm{vec}\big(\widehat{\boldsymbol{C}}^M\big) = \left[\Omega\left(\boldsymbol{f}_{1:t}, \boldsymbol{f}_{1:t}\right) \otimes \boldsymbol{I}_n\right]^{-1} \mathrm{vec}\left[\Omega\left(\boldsymbol{y}^M_{1:t}, \boldsymbol{x}^M_{1:t}\right)\right],$$

where

$$\Omega\left(\boldsymbol{f}_{1:t}, \boldsymbol{f}_{1:t}\right) \otimes \boldsymbol{I}_n = \sum_{s=1}^{t} \Big\{ \Big[\boldsymbol{f}_{s|t} (\boldsymbol{f}_{s|t})^T + \mathrm{Var}\big(\boldsymbol{f}_s \,|\, \boldsymbol{y}^*_1, \cdots, \boldsymbol{y}^*_t; \widehat{\boldsymbol{\theta}}\big) \Big] \otimes \boldsymbol{I}_n \Big\},$$

and

$$\Omega\left(\boldsymbol{y}^M_{1:t}, \boldsymbol{x}^M_{1:t}\right) = \sum_{s=1}^{t} \Big\{ \boldsymbol{y}^M_s (\boldsymbol{f}_{s|t})^T - \Big[\boldsymbol{s}^M_{s|t} (\boldsymbol{f}_{s|t})^T + \mathrm{Cov}\big(\boldsymbol{s}^M_s, \boldsymbol{f}_s \,|\, \boldsymbol{y}^*_1, \cdots, \boldsymbol{y}^*_t; \widehat{\boldsymbol{\theta}}\big) \Big] \Big\},$$

with $\otimes$ denoting the Kronecker product and n denoting the number of monthly variables. Further, we update $\boldsymbol{R}^M$ by letting

$$\widehat{\boldsymbol{R}}^M = \frac{1}{t}\sum_{s=1}^{t}\left[\boldsymbol{e}_{s|t}^M\left(\boldsymbol{e}_{s|t}^M\right)^T + \widehat{\boldsymbol{C}}^M \mathrm{Var}\big(\boldsymbol{x}_s^M \,|\, \boldsymbol{y}_1^*, \cdots, \boldsymbol{y}_t^*; \widehat{\boldsymbol{\theta}}\big)\big(\widehat{\boldsymbol{C}}^M\big)^T\right],$$

where $\boldsymbol{e}_{s|t}^M = \boldsymbol{y}_s^M - \widehat{\boldsymbol{C}}^M \boldsymbol{x}_{s|t}^M$.

Step 3. Update $\boldsymbol{C}^Q$ and $\boldsymbol{R}^Q$: $\boldsymbol{C}^Q$ and $\boldsymbol{R}^Q$ are updated in a manner similar to that of updating $\boldsymbol{C}^M$ and $\boldsymbol{R}^M$.
