# Supplementary material for: Composite GDP nowcasting using macroeconomic variables and electricity data
Source: PLoS One. 2025 Jun 9;20(6):e0324381. doi: 10.1371/journal.pone.0324381 (PMC12148135; doi:10.1371/journal.pone.0324381)
Supplement: S2 Appendix — (PDF) [file pone.0324381.s002.pdf]

S2 Appendix. Prediction in DFM.

When we have obtained the estimated model parameters $\widehat{\boldsymbol{\theta}} = \left(\widehat{\gamma}, \widehat{\boldsymbol{\alpha}}^Q, \widehat{\boldsymbol{\alpha}}^M, \widehat{\Sigma}_u, \widehat{\Sigma}_a^Q, \widehat{\Sigma}_a^M, \widehat{\boldsymbol{C}}^Q, \widehat{\boldsymbol{R}}^Q, \widehat{\boldsymbol{C}}^M, \widehat{\boldsymbol{R}}^M\right)$ using the algorithm detailed in S1 Appendix, we can utilize the Kalman filter to predict $y_{1,3l}^Q$, the year-on-year growth rate of GDP in quarter l, at times $t = 3l - 2$, $t = 3l - 1$, and $t = 3l$. Specifically, we have

$$\begin{aligned} \widehat{y}_{1,3l|3l-2}^{Q,DFM} &= E\left(y_{1,3l}^Q \,|\, \boldsymbol{y}_1^*, \cdots, \boldsymbol{y}_{3l-2}^*; \widehat{\boldsymbol{\theta}}\right) \\ &= \widehat{C}_1 \cdot \widehat{A} \cdot \widehat{A} \cdot E\left(\boldsymbol{x}_{3l-2} \,|\, \boldsymbol{y}_1^*, \cdots, \boldsymbol{y}_{3l-2}^*; \widehat{\boldsymbol{\theta}}\right) = \widehat{C}_1 \cdot \widehat{A} \cdot \widehat{A} \cdot \boldsymbol{x}_{3l-2|3l-2}. \end{aligned}$$

$$\begin{aligned} \widehat{y}_{1,3l|3l-1}^{Q,DFM} &= E\left(y_{1,3l}^Q \,|\, \boldsymbol{y}_1^*, \cdots, \boldsymbol{y}_{3l-1}^*; \widehat{\boldsymbol{\theta}}\right) \\ &= \widehat{C}_1 \cdot \widehat{A} \cdot E\left(\boldsymbol{x}_{3l-1} \,|\, \boldsymbol{y}_1^*, \cdots, \boldsymbol{y}_{3l-1}^*; \widehat{\boldsymbol{\theta}}\right) = \widehat{C}_1 \cdot \widehat{A} \cdot \boldsymbol{x}_{3l-1|3l-1}, \end{aligned}$$

and

$$\begin{aligned} \widehat{y}_{1,3l|3l}^{Q,DFM} &= E\left(y_{1,3l}^Q \,|\, \boldsymbol{y}_1^*, \cdots, \boldsymbol{y}_{3l-1}^*, \boldsymbol{y}_{3l}^M, y_{3l,2}^Q, \cdots, y_{3l,m}^Q; \widehat{\boldsymbol{\theta}}\right) \\ &= \widehat{C}_1 \cdot E\left(\boldsymbol{x}_{3l} \,|\, \boldsymbol{y}_1^*, \cdots, \boldsymbol{y}_{3l-1}^*, \boldsymbol{y}_{3l}^M, y_{3l,2}^Q, \cdots, y_{3l,m}^Q; \widehat{\boldsymbol{\theta}}\right), \end{aligned}$$

where $\widehat{C}_1$ denotes the first row vector of $\widehat{C}$. Here, $\boldsymbol{x}_{3l-2|3l-2}$, $\boldsymbol{x}_{3l-1|3l-1}$, and $E\left(\boldsymbol{x}_{3l} \,|\, \boldsymbol{y}_1^*, \cdots, \boldsymbol{y}_{3l-1}^*, \boldsymbol{y}_{3l}^M, y_{3l,2}^Q, \cdots, y_{3l,m}^Q; \widehat{\boldsymbol{\theta}}\right)$ can be obtained using Step 1 of the E-step presented in S1 Appendix.
